# Gene co-expression network analysis reveals coordinated regulation of three characteristic secondary biosynthetic pathways in tea plant (*Camellia sinensis*)

**DOI:** 10.1186/s12864-018-4999-9

**Published:** 2018-08-15

**Authors:** Yuling Tai, Chun Liu, Shuwei Yu, Hua Yang, Jiameng Sun, Chunxiao Guo, Bei Huang, Zhaoye Liu, Yi Yuan, Enhua Xia, Chaoling Wei, Xiaochun Wan

**Affiliations:** 1 School of Life Science, Anhui Agricultural University, Hefei, 230036 China; 2 State Key Laboratory of Tea Plant Biology and Utilization, Anhui Agricultural University, Hefei, 230036 China; 3 BGI Genomics, BGI-Shenzhen, Shenzhen, 518083 China

**Keywords:** Tea plant, Co-expression network analysis, Characteristic metabolites, RNA-seq, Secondary metabolic pathway

## Abstract

**Background:**

The leaves of tea plants (*Camellia sinensis*) are used to produce tea, which is one of the most popular beverages consumed worldwide. The nutritional value and health benefits of tea are mainly related to three abundant characteristic metabolites; catechins, theanine and caffeine. Weighted gene co-expression network analysis (WGCNA) is a powerful system for investigating correlations between genes, identifying modules among highly correlated genes, and relating modules to phenotypic traits based on gene expression profiling. Currently, relatively little is known about the regulatory mechanisms and correlations between these three secondary metabolic pathways at the omics level in tea.

**Results:**

In this study, levels of the three secondary metabolites in ten different tissues of tea plants were determined, 87,319 high-quality unigenes were assembled, and 55,607 differentially expressed genes (DEGs) were identified by pairwise comparison. The resultant co-expression network included 35 co-expression modules, of which 20 modules were significantly associated with the biosynthesis of catechins, theanine and caffeine. Furthermore, we identified several hub genes related to these three metabolic pathways, and analysed their regulatory relationships using RNA-Seq data. The results showed that these hub genes are regulated by genes involved in all three metabolic pathways, and they regulate the biosynthesis of all three metabolites. It is notable that light was identified as an important regulator for the biosynthesis of catechins.

**Conclusion:**

Our integrated omics-level WGCNA analysis provides novel insights into the potential regulatory mechanisms of catechins, theanine and caffeine metabolism, and the identified hub genes provide an important reference for further research on the molecular biology of tea plants.

**Electronic supplementary material:**

The online version of this article (10.1186/s12864-018-4999-9) contains supplementary material, which is available to authorized users.

## Background

Tea, produced from the leaves of the tea plant, *Camellia sinensis* (L.), belonging to family *Theaceae*, is one of the most popular natural non-alcoholic beverages consumed worldwide. To date, nearly 4000 bioactive compounds have been identified in tea [1] including catechins, caffeine, theanine and volatile oils [2]. Catechins generally contain six different monomers, namely catechin (C), gallocatechin (GC), epicatechin (EC), epigallocatechin (EGC), epicatechin gallate (ECG) and epigallocatechin gallate (EGCG) [3]. Catechins, caffeine and theanine are the main three characteristic biologically active compounds in tea [4]. They are not only important contributors to flavour, but also have beneficial effects on human health due to their autoxidation and anticancer activity [5] and their ability to lower blood pressure [6], prevent cardiovascular diseases [7], and assist weight loss [8].

Gene co-expression network analysis (GCNA) is a genetic approach for analysing correlations between genes using large-scale gene expression profiling data that is especially useful for investigating relationships between functional modules and phenotypic traits [9, 10]. Weighted GCNA (WGCNA) is one of the most popular GCNA-based approaches, and this correlation-based technique describes and visualises co-expression networks between genes using transcriptomic data [11]. This technique has been successfully utilized to identify the gene modules in *Arabidopsis* and rice that are related to drought and bacterial stress [12]. Module assignment in WGCNA is a flexible process which reduces the complexity of a dataset from hundreds of genes to a smaller number of modules.

Researchers have focused on the molecular mechanisms involved in plant growth, development [13, 14] and the production of secondary metabolites [15] in tea plants. Regulatory mechanisms underlying secondary metabolite biosynthesis, particularly those related to catechins, theanine and caffeine, have been explored at the molecular level. Recent advances in next-generation sequencing of RNA [16] have been accompanied by an increase in the amount of available transcriptomic data from different tissues of tea plants [17], from different species of the genus *Camellia* [18], and from plants grown under different stress conditions [19, 20]. Most research has focused on using RNA-Seq data from tea plants to reveal the regulatory mechanisms and relationships between gene expression and production of the characteristic secondary metabolites. Li et al. constructed a possible transcription factor regulation network of flavonoid, caffeine and theanine biosynthesis using 13 different samples from tea plants (various organs and developmental stages) through correlation analysis [21]. However, relatively few studies have investigated co-expression networks in tea plants using RNA-Seq data. In the present study, WGCNA was performed using RNA-Seq data from ten tissues, and modules significantly correlated with the three characteristic compounds were identified and analysed. Furthermore, highly-connected hub genes related to these modules were identified. This novel approach revealed the regulatory mechanisms of characteristic metabolic pathways in tea plants, and highlighted the important role of light in the biosynthesis of catechins, theanine and caffeine.

## Results

### Determination of catechins, theanine and caffeine content

**Fig. 1:**
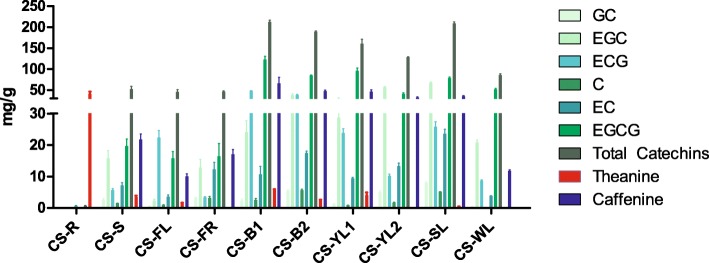
Content of catechins, theanine and caffeine in different tissues of *Camellia sinensis*. Tissues include apical buds in May (CS-B1) and June (CS-B2), first young leaves (CS-YL1), second young leaves (CS-YL2) and mature leaves in summer (CS-SL), mature leaves in winter (CS-WL), stems (CS-S), flowers (CS-FL), fruits (CS_FR) and roots (CS-R)

High-performance liquid chromatograph (HPLC) analysis was used to determine the content of catechins (C, EC, GC, EGC, ECG and EGCG), theanine and caffeine in ten different tissues from *C. sinensis* cv. *Shuchazao*. (Fig. 1). The results indicated that accumulation of individual catechin compounds and caffeine varied between different tissues and seasons. The total amounts of catechins and caffeine were greater in tender shoots than other tissues, especially in buds and first leaves (> 200 mg/g). Galloylated catechins such as ECG and EGCG were the predominant characteristic phenolic compounds, and reached 48.3 and 122.5 mg/g, respectively. Similarly, the caffeine content was significantly higher in tender shoots, but variation between new and old shoots was less than for catechins. By contrast, total catechins and caffeine were much less abundant in roots than in other tissues (0.67 and 0.0029 mg/g, respectively). However, theanine, an important compound in tea, reached 40.8 mg/g in roots, which was 6-fold higher than in buds.

### De novo assembly and functional annotation of the *C. sinensis* transcriptome

We obtained 111 gigabases of sequencing data (average 11 gigabase/sample) from ten samples. De novo assembly of the *C. sinensis* transcriptome was performed using the Trinity package [22], and 91,494 unigenes were obtained after removing short contigs (≤200 bp) and redundancy by TGICL [23]. In order to reduce potential assembly errors, we filtered unigenes with a Fragments Per Kilobase per Million mapped fragments (FPKM) value less than 0.3 in at least eight of the ten tissues [24]. Finally, a high-quality transcriptomic library of 87,319 unigenes with an N50 of 1406 bp and an average length of 874 bp was obtained. Functional annotation successfully aligned 54,827 (62.79%), 58,770 (67.30%), 40,700 (46.61%), 43,910 (50.29%), 25,172 (28.83%) unigenes to the NT (Non-redundant nucleotide database), NR (Non-redundant protein database), Swiss-Prot (Annotated protein sequence database), KEGG (Kyoto Encyclopaedia of Genes and Genomes), and GO (Gene Ontology) databases, respectively. Overall, 72.68% (63,464 of 87,319) of unigenes were annotated (Table 1).

**Table 1:** Summary of sequence assembly and functional annotation

Level	Item	No. of sequences	Percentage (%)
Assembly	Total number of Unigenes	87,319	–
	Total bases (Mb)	76,328,365	–
	N50 (bp)	1406	–
	Average Unigene length (bp)	874	–
Annotation	NT-annotated	54,827	62.79
	NR-annotated	58,770	67.30
	SwissProt-annotated	40,700	46.61
	KEGG-annotated	43,910	50.29
	COG-annotated	22,252	25.48
	GO-annotated	25,172	28.83
	All annotated Unigenes	63,464	72.68

**Fig. 2:**
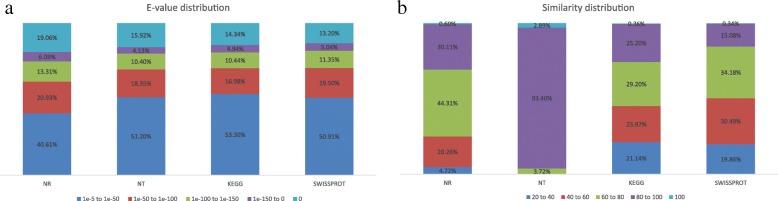
E-value and sequence similarity analysis of unigenes against NR (Non-redundant protein), Nt (Non-redundant nucleotide), KEGG (Kyoto Encyclopedia of Genes and Genomes) and Swiss-Prot (Annotated protein sequence) databases. **a** E-value distribution of BLAST hits for each unigene with a cutoff E-value of 1.0E-5. **b** Similarity distribution of the top BLAST hits for each unigene

The E-value distribution of the top hits in the NR, NT, KEGG and Swiss-Prot databases shows that an average of 51% of mapped sequences shared significant homology (< 1.0E^− 50^), and nearly 41.99% of sequences shared greater than 80% similarity. These results confirmed the high quality of the assembled unigenes in the transcriptomic library (Fig. 2).

### Analysis of gene expression and identification of differentially expressed genes (DEGs)

**Fig. 3:**
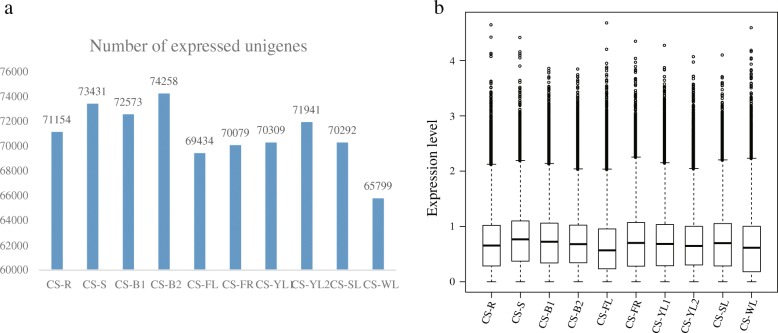
Number of expressed unigenes and their expression levels in different tissues of *C. sinensis*. **a** Number of unigenes expressed in each tissue (given above bars). **b** Expression levels of unigenes in the ten tissues. Expression levels of all genes were evaluated using log10-transformed FPKM values

Clean reads from ten different *C. sinensis* tissues were mapped to the high-quality unigene sets using Bowtie2 [25]. Expression levels of unigenes were calculated in each tissue using FPKM values, and unigenes with an FPKM ≥0.3 [26] were defined as expressed. The number of genes expressed in each tissue ranged from 65,799 to 74,258, with an average FPKM of 16.47 (Fig. 3). A total of 55,607 DEGs were identified by in pairwise comparison for further analysis.

### Construction of the gene co-expression network

We constructed an unassigned network using DEGs identified by pairwise comparison from the ten tissues [27, 28]. All tissues were clustered initially without any outlier tissues (Additional file 1). A scale-free topology model with a soft threshold of 30 was used to ensure that the network was biologically relevant, resulting in 53,279 (95.81%) out of 55,607 unigenes parsed into 35 co-expression modules, with the module size ranging from 55 to 9041.

### Identification of content-related modules

**Fig. 4:**
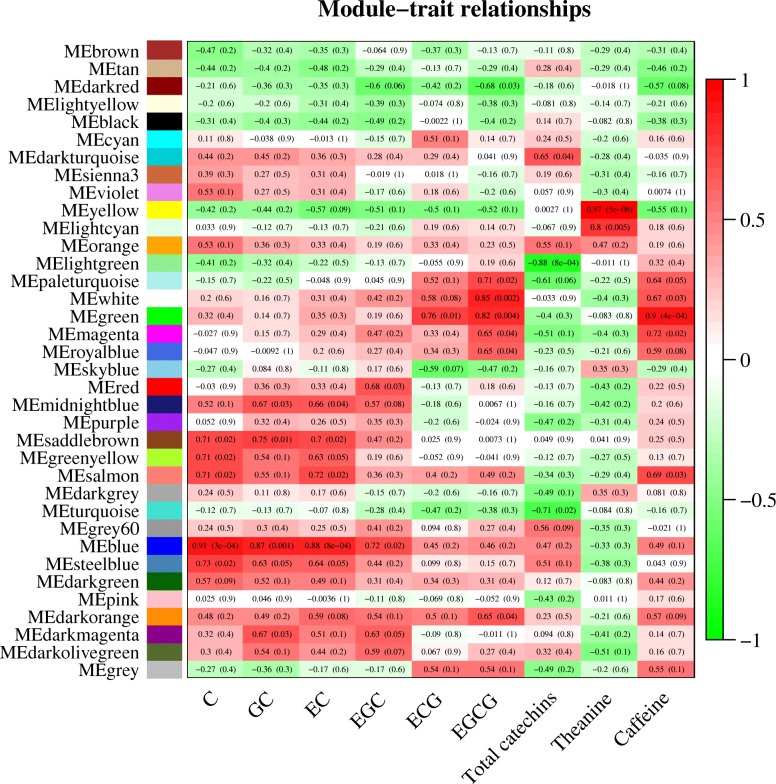
Module-trait relationships. Each row corresponds to a module eigengene (correlation between a column and a trait). Each cell contains the corresponding correlation and *p*-value. The table is colour-coded by correlation in accordance with the figure

We investigated correlations between the characteristic components (C, GC, EC, EGC, ECG, EGCG, theanine and caffeine) of tea and the 35 co-expression modules. We identified 20 modules that were significantly (*p* < 0.05) correlated with characteristic components (content-related modules), with highly positive coefficients for modules associated with C (0.91), GC (0.87), EC (0.88), EGC (0.72), ECG (0.76), EGCG (0.85), theanine (0.97) and caffeine (0.9) coloured blue, blue, blue, blue, green, white, yellow and green, respectively. In addition, EGCG was significantly negatively correlated with the dark red module (− 0.68), while total catechins were negatively correlated with the light green module (− 0.88), turquoise module (− 0.71) and pale turquoise module (− 0.61). Importantly, there was only two modules (yellow and light cyan) significantly associated with theanine (Fig. 4 and Additional file 2).

### Functional annotation and enrichment of content-related modules

**Fig. 5:**
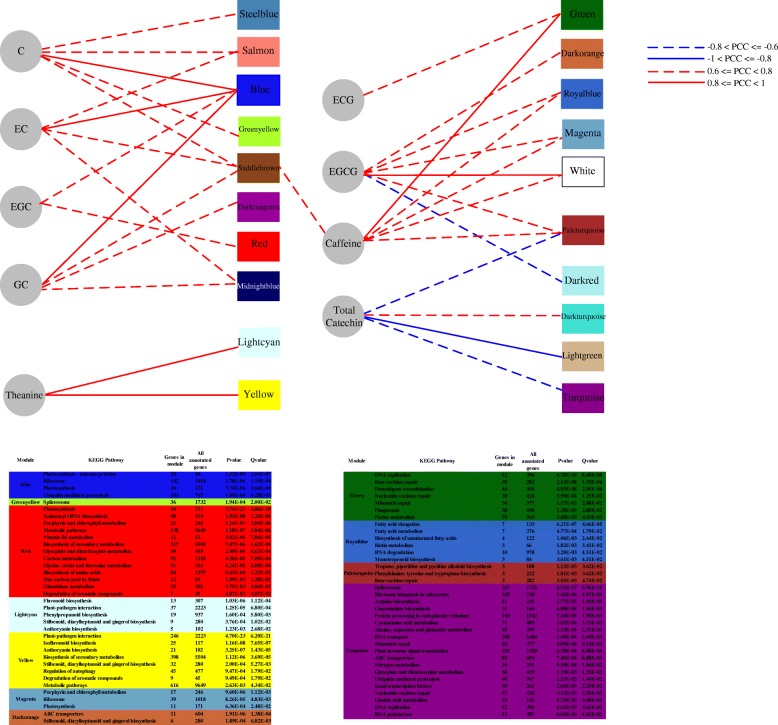
KEGG functional enrichment analysis of co-expression modules associated with phenotypic traits. The top panel shows correlations between modules and traits, while the bottom panel indicates the KEGG functional enrichment analysis of each trait-related module

To identify the biological roles of modules associated with catechins, theanine and caffeine, functional annotation and enrichment of these modules were analysed by KEGG pathway analysis. Detailed functional enrichment information from KEGG pathway annotation related to these modules (Q-value < 0.05) is shown in Fig. 5. According to KEGG pathway enrichment analysis, unigenes in content-related modules were enriched in different metabolic pathways, especially those related to characteristic metabolites in tea. For example, ‘Biosynthesis of amino acids’ was enriched in red modules with EGC, and ‘Isoflavonoid biosynthesis’, ‘Anthocyanin biosynthesis’, ‘Flavonoid biosynthesis’, ‘Phenylpropanoid biosynthesis’ and ‘Degradation of aromatic compounds’ were enriched in the yellow and light cyan modules associated with theanine. ‘Purine metabolism’ and ‘Tropane, piperidine and pyridine alkaloid biosynthesis’ were enriched in the green and magenta modules associated with EGCG and caffeine. ‘Nitrogen metabolism’, ‘Arginine biosynthesis’ and ‘Alanine, aspartate and glutamate metabolism’ were enriched in the turquoise module associated with total catechins.

### Functional analysis of unigenes related to photosynthesis

Unigenes in blue, red and magenta modules were significantly enriched in ‘Photosynthesis’ according to KEGG pathway enrichment analysis. Further functional analysis indicated that these unigenes were associated with ferredoxin, photosystem II, photosystem I, ferredoxin--NADP+ reductase, F-type H + −transporting ATPase subunit delta, and some other categories (Additional file 3). Light is an important environmental parameter that drives photosynthesis, and photosynthesis might influence catechin biosynthesis via the provision of carbon sources.

### Hub gene identification and visualisation

**Fig. 6:**
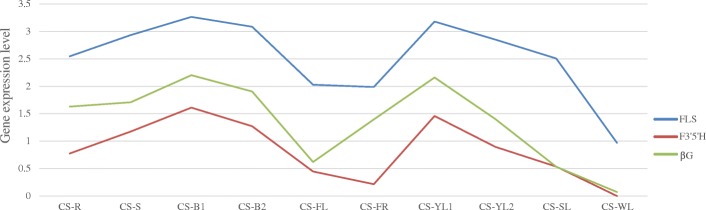
Gene expression patterns of F3’5’H, FLS and βG in the green module. Expression levels of F3’5’H, FLS and βG genes are shown using log10-transformed FPKM values

Hub genes in modules may be more important than other genes in the network, and they can be considered representative of the module in the biology network. Detailed information on all hub genes of each content-related module is listed in Additional file 4. Hub gene analysis identified WD40 repeat, ethylene-responsive transcription factor, MYB, WRKY and bHLH in the blue module. Heat shock proteins in the blue module were also identified, as were ABC transporters in yellow and light cyan modules. The green module contains flavonoid 3’,5’-hydroxylase (*F3’5’H*), flavonol synthase (*FLS*) and beta-glucosidase (*βG*). The yellow module includes two glutamine synthetase (*GS*) genes, as well as shikimate O-hydroxycinnamoyltransferase (*HCT*) and UDP-glycosyltransferase. The correlation coefficient between F3’5’H and ECG, and EGCG and caffeine was more than 0.76 (*p*-value < 0.05), and the correlation coefficient between *βG* and ECG, and EGCG and caffeine was more than 0.67 (*p*-value < 0.05). However, two GS genes were not significantly correlated with catechins and caffeine, although they were significantly correlated with theanine (correlation coefficient > 0.98, *p*-value < 0.001; Fig. 6 and Table 2).

**Table 2:** Correlation analysis of gene expression related to caffeine and catechins

Gene ID	Correlation coefficient				*p*-value			
	Caffeine	ECG	EGCG	Theanine	Caffeine	ECG	EGCG	Theanine
F3’5’H_Unigene20838	0.86	0.76	0.81	–	0.001483	0.01045	0.004592	–
FLS_Unigene16195	0.88	0.76	0.83	–	0.000645	0.01005	0.003184	–
βG_CL6189.Contig3	0.79	0.67	0.76	–	0.006371	0.03351	0.0110702	–
GS_Unigene22464	–	–	–	0.98275	–	–	–	0.00000038
GS_CL1326.Contig1	–	–	–	0.98167	–	–	–	0.000000483

**Fig. 7:**
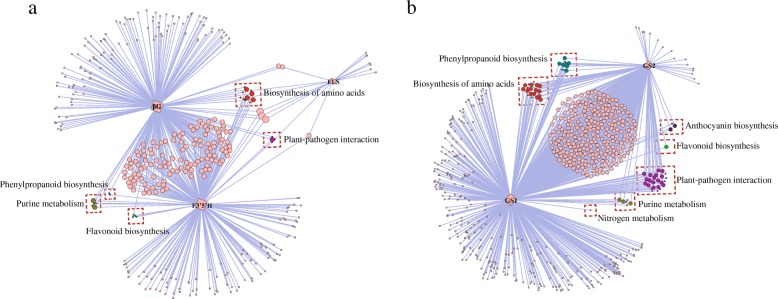
Genes highly co-expressed with genes involved in catechin and theanine pathways. **a** Genes highly co-expressed with F3’5’H, FLS and βG in the catechin pathway. **b** Genes highly co-expressed with GS in the theanine pathway. Coloured circles represent genes, and edges represent correlations among genes

Genes highly co-expressed with *F3’5’H*, *FLS*, *βG* and *GS*s are shown in Fig. 7. Many more genes were co-expressed with *F3’5’H* and *βG* than with *FLS*. We also found five genes involved in ‘Purine metabolism’ that were co-expressed with *F3’5’H* and *βG*, and 11 genes involved in ‘Biosynthesis of amino acids’ that were co-expressed with *F3’5’H*, *βG* and *FLS* (Fig. 7a). A number of genes were co-expressed with *GS*s, along with ten, one and three genes involved in ‘Phenylpropanoid biosynthesis, ‘Flavonoid biosynthesis’ and ‘Anthocyanin biosynthesis’, respectively. We also identified six genes involved in ‘Purine metabolism’ (Fig. 7b).

### Validation of unigenes by qPCR

**Fig. 8:**
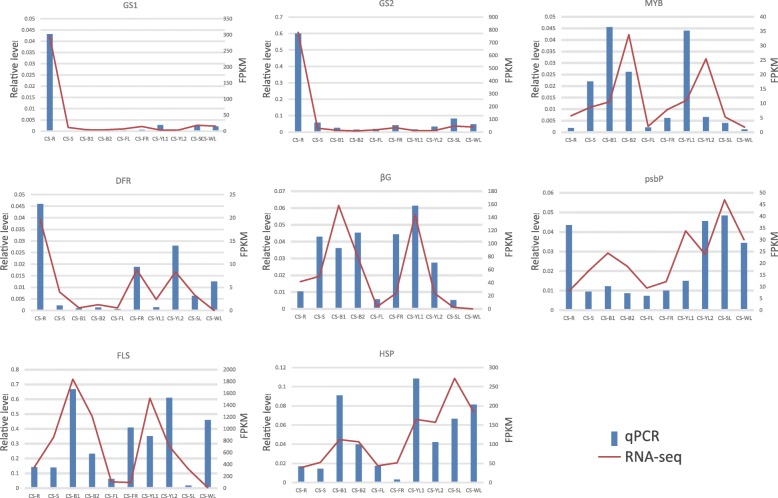
Validation of the expression of candidate unigenes by qPCR. Gene expression levels were determined by qPCR and are presented as mean ± SD values calculated by the 2^ΔCt^ method

In order to confirm the accuracy of unigene expression levels, eight unigenes from 18 content-related modules were selected for qPCR analysis, and their relative expression levels were compared with FPKM values from RNA-Seq data. The results showed that expression of all eight unigenes measured by qPCR was consistent with the RNA-Seq data. Of the eight unigenes, correlation analysis between FPKM values and qPCR data showed that three had a correlation coefficient > 0.9, and three had a correlation coefficient > 0.7 (Fig. 8).

## Discussion

We constructed a *C. sinensis* gene co-expression network using a WGCNA approach and identified co-expression modules using transcriptome data from ten tissues. Correlation analysis between co-expression modules and three characteristic metabolites (catechins, theanine and caffeine) was carried out, and 20 significant content-related modules (*p*-value < 0.05) and 6 highly significant content-related modules (correlation coefficient ≥ 0.8 and *p*-value < 0.05) were identified. These modules consist of highly connected functional genes, and different modules appear to be involved in individual functions [29]. Meanwhile, KEGG pathway enrichment analysis of modules associated with catechins, theanine and caffeine indicated that the three characteristic secondary pathways in tea plants are related to each other at the transcriptomic level. The results also indicated that one component can be regulated by multiple modules, and one module can simultaneously be associated with multiple components (Fig. 5).

Amino acid metabolism-related pathways were also found to be enriched in modules associated with catechins. For instance, ‘Glycine, serine and threonine metabolism’, and ‘Biosynthesis of amino acids’ were significantly enriched in the red module, which was highly positively correlated with EGC. ‘Arginine biosynthesis’, ‘Alanine, aspartate and glutamate metabolism’, and ‘Nitrogen metabolism’, were significantly enriched in the turquoise module, which was highly negatively correlated with total catechins. ‘Carbon metabolism’ was significantly enriched in the red module. Pathways enriched in the red and turquoise modules suggest that carbon and amino acid metabolism may have an important influence on EGC, EGCG and total catechins. Unigenes in the yellow and light cyan modules, which were significantly associated with theanine, were enriched in pathways involved in catechin biosynthesis such as ‘Flavonoid biosynthesis’, ‘Anthocyanin biosynthesis’ and ‘Isoflavonoid biosynthesis’. These results indicate that biosynthesis of theanine is highly correlated with catechins, which suggests that theanine might play a vital role in the biosynthesis of catechins as unknown precursors. This result is consistent with the work of Feldheim et al. [30] who monitored the turnover of theanine in tea by investigating the distribution of isotopically labelled N-ethyl theanine in tea seedlings and young shoots. They found that the N-ethyl group of theanine was incorporated into the phloroglucinol nucleus of catechins. Similarly, Tanaka et al. [31] demonstrated that theanine is degraded to a Strecker aldehyde and conjugated with polyphenol rings to generate a novel polyphenol, ethylpyrrolidinonyl theasinensin, during the production of black tea.

In plants, nucleotides can be derived de novo from 5-phosphoribosyl-1-pyrophosphate and various simple molecules, but they can also be synthesised from preformed nucleosides and nucleobases via salvage reactions [32]. The de novo pathway of purine and pyrimidine biosynthesis is constitutive, but salvage enzymes may perform a special role in the activation of resting cells and in the response to environmental changes [33]. The green module was positively correlated with caffeine, ECG and EGCG. Based on KEGG pathway enrichment analysis, ‘Purine metabolism’, ‘Nucleotide excision repair’, ‘DNA replication’, ‘Homologous recombination’ and ‘Base excision repair’ were significantly enriched, suggesting salvage reactions, rather than the de novo pathway, may play a more important role in the biosynthesis of caffeine.

Module hub genes are generally considered representative of a given module in a biological network. Previous studies reported that MYB-bHLH-WDR (MBW) ternary complexes comprise the essential regulatory machinery for catechin and anthocyanin biosynthesis [34, 35]. In the present study, transcription factors MYB, bHLH, WD40, WRKY, and zinc finger were identified as hub genes in modules related to C, GC, EC and EGC. In addition, three genes involved in flavonoid biosynthesis (*F3’5’H, FLS* and *βG*) were identified in modules related to ECG, ECGC and caffeine, along with two genes involved in theanine biosynthesis (*GS*) in a module related to theanine. Flavonoid-3’5’-hydroxylase (*F3’5’H*), which belongs to the cytochrome P450 family, is the key enzyme related to anthocyanin biosynthesis [36]. Galloylated catechins such as ECG or EGCG are produced by ECGT from the substrates βG and nongalloylated catechins EC or EGC [37]. Correlation analysis between *F3’5’H*, *FLS*, *βG*, catechins and caffeine showed that *F3’5’H*, *FLS* and *βG* were significantly positively correlated not only with ECG and EGCG, but also caffeine, which indicates the existence of a regulatory relationship between catechin and caffeine pathways. Genes highly co-expressed with *F3’5’H*, *βG* and *FLS* were also involved in purine metabolism and biosynthesis of amino acids, while genes highly co-expressed with *GS*s were also involved in phenylpropanoid biosynthesis, flavonoid biosynthesis, anthocyanin biosynthesis and purine metabolism.

Tea is a sciophilous plant adapted to the understorey of tropical rainforests that possesses numerous inducible physiological adaptations protecting against light-associated damage. In previous studies, researchers found that shade treatment can effectively improve the quality of tea beverages [38], and the leaves of tea plants grown in the shade contain higher amino acid levels and a lower catechin content [39]. Furthermore, shade treatment can effectively reduce the biosynthesis of flavonoids and lignins by reducing the expression of genes in the flavonoid pathway [40]. Researchers [41] cloned a novel CsDFR gene that actively responds to light treatment, and showed that light might be effective for activating the biosynthesis of phenylpropanoids that protect against light stimuli. Recently, Tai et al. [42] analysed the promoters of *LAR, TCS* and *TS* in a tea BAC library, and identified numerous light-responsive cis-acting elements in *LAR, TCS* and *TS* genes. Light is an important environmental parameter that drives photosynthesis, and it might regulate genes related to the catechin biosynthesis pathway [40, 41]. In the present study, we found that photosynthesis-related unigenes were significantly enriched in modules positively associated with C, GC, EC, EGC and EGCG based on KEGG pathway enrichment analysis. These findings strongly indicate that photosynthesis might influence catechin synthesis via provision of carbon sources. Further investigations are clearly required to uncover the relationship between light and the biosynthesis of catechins.

## Conclusion

We analysed the content of catechins, theanine and caffeine in ten different tissues from tea plants, and constructed a co-expression network to investigate relationships between genes and these three characteristic metabolites. The results indicated that genes related to catechins, theanine and caffeine were influenced by each other, especially key genes associated with the metabolic pathways of these characteristic compounds. Furthermore, light was identified as an important factor in the biosynthesis of catechins. WGCNA proved to be a novel method for analysing the connection between metabolites and gene expression. This method holds potential for further exploration of large-scale transcriptomic data.

## Methods

### Plant material

Six-year-old tea plants (*C. sinensis* L. O. Kuntze cv. *Shuchazao*) were used in this study. The field experiment was performed in a typical tea-producing garden at De Chang Fabrication Base in Anhui Province, China (Shucheng, latitude 31.3 N, longitude 117.2E above sea level) under natural conditions. Tea plants were grown in an experimental plot with 150 cm between rows and 40 cm between plants within a row, and yellow brown acidic soil was employed. Tea plants were divided into three groups, each group consisted three rows, with at least 100 plants pooled per group, and samples were randomly selected from these three groups, with no fewer than 6−10 samples obtained from ten different tissues of tea plants. The maximum air temperature in the tea garden was about 27°C in the daytime and a minimum of 21°C at night during May and June, and ~ 27°C in the daytime and a minimum of 21°C at night in December. Apical buds (CS-B1) in May, apical buds (CS-B2) in June, first young leaves (CS-YL1) in June, second young leaves (CS-YL2) in June, mature leaves in summer (CS-SL) in June, stem (CS-S), mature leaves in winter (CS-WL) in December, flowers (CS-FL), fruits (CS-FR) in June, and roots (CS-R) in June comprised the ten different tissue samples studied. Tea plants were watered and fertilised equivalently, and tea plants with uniform height and crown breadth, and without signs of disease and insects, were selected for experiments. Three biological replicates were performed for each sample. All samples were immediately frozen in liquid nitrogen, and stored at − 80°C until RNA extraction.

### Extraction and HPLC determination of catechins, theanine and caffeine

Catechins, theanine and caffeine were extracted from samples as described previously [18] with some modifications. Catechins and caffeine were extracted with 80% methanol, while theanine was extracted with hot water as previously described [43]. The obtained supernatants (catechins, theanine and caffeine) were filtered through a 0.22 μm membrane prior to HPLC analysis. All samples were analysed using three biological repeats. The catechin and caffeine content was determined using a Waters 2695 HPLC system (Waters, USA). The column temperature was set to 25°C, and the detection wavelength was 278 nm. The theanine content was measured using a Waters 600E series HPLC system (Waters, USA) at a detection wavelength of 199 nm [44]. The mobile phase ratio and injection approach were as described previously [18]. C, GC, EC, EGC, ECG, EGCG, theanine and caffeine standards were purchased from Shanghai Winherb Medical Technology, Ltd., China.

### RNA isolation, transcriptomic library construction and RNA-Seq

Total RNA was extracted separately from the ten tissues using a modified CTAB (cetyltrimethyl ammonium bromide) method with three biological replicates [45]. The yield and quality of RNA were determined by agarose gel electrophoresis (AGE) and a Nanodrop 2000 instrument. RNA samples with an A260/A280 > 1.8, A260/A230 > 1.8, and RNA integrity number (RIN) > 8 were considered acceptable for library construction. Equal amounts of RNA from three different samples were pooled before cDNA library preparation, and mRNA enrichment, cDNA synthesis, fragmentation, adapter addition, selection of fragment size, PCR amplification, and transcriptomic sequencing were performed by staff at the Beijing Genome Institute (BGI; Shenzhen, China) as previously described by Liu et al. [46] and Gu et al. [47]. Briefly, mRNAs were purified from total RNA using magnetic beads with Oligo (dT) and cleaved into short sequences. First-strand cDNA synthesis was then performed with random primers (TaKaRa, Japan), and double-stranded cDNAs were then prepared using these short fragments as templates. Adapters were ligated to the short fragments using T4 DNA ligase (Invitrogen, USA), and after end repair and ligation of adapters, products were enriched by PCR to generate the cDNA library. The cDNA library was examined using an Agilent 2100 Bioanalyzer prior to sequencing on an Illumina HiSeq 2000 sequencing platform [48]. Paired-end reads were generated with a length of 90 bp for each read.

### Data preprocessing and de novo assembly

Raw sequencing reads were subjected to preliminary screening to remove low-quality reads and reads with adaptor sequences using the filter command in SOAPnuke (version 1.5.6) with the low-quality threshold set to 10 [49]. We obtained 111 gigabases (average 11 gigabase/sample) from ten samples, and in order to reduce the assembly error, remaining reads were then de novo assembled using the Trinity package (release-20,130,225) with parameters ‘--min_glue 3 and --min_kmer_cov 3’. These assembly parameters were used previously by Shao et al. [50] and Li et al. [51]. Assembled unigenes were defined after removing short and redundant contigs (≤200 bp) with TGICL software (version 2.1) [23] from the Trinity assembly. Final assembled datasets were defined by removing unigenes with FPKM values less than 0.3 based on the work of Ramskold et al. [26], as applied in several other studies including Łabaj et al. [52] and Sam et al. [53]

### Annotation and classification of unigenes

All assembled non-redundant and filtered unigenes were annotated by alignment to the NR database, the Swiss-Prot protein database, and the COG database using BLASTX with an E-value cut-off of 1×10^− 5^ [54]. Meanwhile, these unigenes were also annotated using the NT database and BLASTN. KEGG metabolic pathway annotation of unigenes was carried out after mapping to the KEGG database [55], which helped to elucidate the complex biological functions of genes. Based on the BLAST results from the NR database, GO annotation was carried out using the Blast 2 GO program (version 2.3.4) [56].

### Identification of gene expression and DEGs

Expression levels of unigenes were calculated using the FPKM method. Firstly, reads were mapped to unigene datasets by Bowtie2 (version 2.1.0, http://bowtie-bio.sourceforge.net/bowtie2/index.shtml) at a sensitive setting. Based on the Bowtie results, FPKM values for each unigene were subsequently calculated by RESM (version 1.2.29) [57] with default parameters. DEGs were identified based on the method described by Audic et al. [58]. Genes with|log2ratio| ≥ 1 and false discovery rate (FDR) < 0.05 were identified as DEGs.

### Construction of gene co-expression networks

Gene co-expression networks were constructed using the WGCNA approach with R packages (version 3.2.2). DEGs expressed in at least one pairwise comparison in ten tissues were retained for co-expression network construction by WGCNA analysis [11]. All tissues were initially clustered to analyse the sample height. Following application of the scale-free topology criterion described previously, a soft threshold of 30 was chosen. Based on the topological overlap-based dissimilarity measure [59], unigenes were first hierarchically clustered, and the gene dendrogram was used for module detection by the dynamic tree cut method (mergeCutHeight = 0.25, minModuleSize = 30). In the weighted gene co-expression network, gene connectivity was based on the edge weight (ranging from 0 to 1) determined by the topology overlap measure, which reflects the strength of the communication between two genes. The weights across all edges of a node were summed and used to define the level of connectivity, and nodes with high connectivity were considered hub genes.

### Identification of content-related modules

To identify modules associated with catechins, theanine and caffeine, we first calculated the module eigengenes of each module, then correlated these with the catechin, theanine and caffeine content using Pearson’s correlation coefficients and an asymptotic confidence interval based on Fisher’s Z transformation. Modules with *p*-values < 0.05 were identified as content-related modules. To further characterise these modules, enrichment of annotated unigenes in each content-related module was investigated using the phyper function within the R platform based on KEGG pathway annotation, and q-value or FDR corrections were applied by multiple testing [60]. We defined KEGG pathways with a q-value or FDR < 0.05 as significantly enriched [61].

### Module hub gene selection and visualisation

The most central and connected genes, involved in numerous interactions, were considered hub genes [62], which are likely to play a more important role in a given module than other genes in the overall co-expression network. In this study, we categorised the top 2% of the most highly connected genes in a module as hub genes based on the size of the module. Co-expression interactions and patterns of hub genes were visualised using Cytoscape [63].

### qPCR validation of selected unigenes

In order to evaluate the assembly quality of RNA-seq data, the expression patterns of eight selected transcripts were monitored by qPCR. RNA samples were isolated from samples using the CTAB method [45], and total RNA was reverse-transcribed into single-stranded cDNAs using a reverse transcription kit for real-time PCR (TaKaRa). Detailed information (unigene IDs and primer sequences) related to the selected transcripts used for qPCR is listed in Additional file 5. PCR amplification was performed according to the manufacturer’s instructions using a CFX96TM real-time PCR system (Bio-Rad) with an annealing temperature of 60 °C. The housekeeping gene glyceraldehyde-3-phosphate dehydrogenase (*GAPDH*) was used as an internal reference gene, and relative expression levels of target genes were calculated using the 2^ΔCt^ method [64]. All qPCRs were analysed using three technical and three biological replicates.

## Additional files

Additional file 1: Clustering dendrogram of samples based on gene expression. (PDF 99 kb)

Additional file 2: Modules significantly (*p* < 0.05) correlated with characteristic components in tea. (XLSX 11 kb)

Additional file 3: Functional analysis of unigenes in blue, red and magenta modules enriched in the photosynthesis pathway. (XLSX 12 kb)

Additional file 4: Hub genes for each highly significant content-related module. (XLSX 54 kb)

Additional file 5: Unigene IDs and primer sequences for selected transcripts used for qPCR validation. (XLSX 9 kb)

## Abbreviations

AGE: Agarose gel electrophoresis
C: Catechin
COG: Clusters of Orthologous Groups of Proteins
CS_FR: Fruit CS-R Root
CS-B1: Apical bud
CS-B2: Apical bud
CS-FL: Flower
CS-S: Stem
CS-SL: Mature leaf in summer
CS-WL: Mature leaf in winter
CS-YL1: First young leaf
CS-YL2: Second young leaf
EC: Epicatechin
ECG: Epicatechin gallate
EGC: epigallocatechin
EGCG: Epigallocatechin gallate
F3’5’H: Flavonoid 3’,5’-hydroxylase
FLS: Flavonol synthase
FPKM: Fragment Per Kilobase of exon model per Million mapped reads
GAPDH: Glyceraldehyde-3-phosphate dehydrogenase
GC: Gallocatechin
GO: Gene Ontology
GS: Glutamine synthetase
HCT: Shikimate O-hydroxycinnamoyl transferase
HPLC: High-performance liquid chromatography
KEGG: Kyoto Encyclopedia of Genes and Genomes
Nr: Non-redundant protein database
Nt: Non-redundant nucleotide database
qPCR: Quantitative real-time polymerase chain reaction
Swiss-Prot: Annotated protein sequence database
WGCNA: Weighted gene co-expression network analysis
βG: Beta-glucosidase
