# Supplementary figures and images for: Gene co-expression network analysis reveals coordinated regulation of three characteristic secondary biosynthetic pathways in tea plant (Camellia sinensis)
Source: BMC Genomics. 2018 Aug 15;19:616. doi: 10.1186/s12864-018-4999-9 (PMC6094456; doi:10.1186/s12864-018-4999-9)

# Cluster Dendrogram

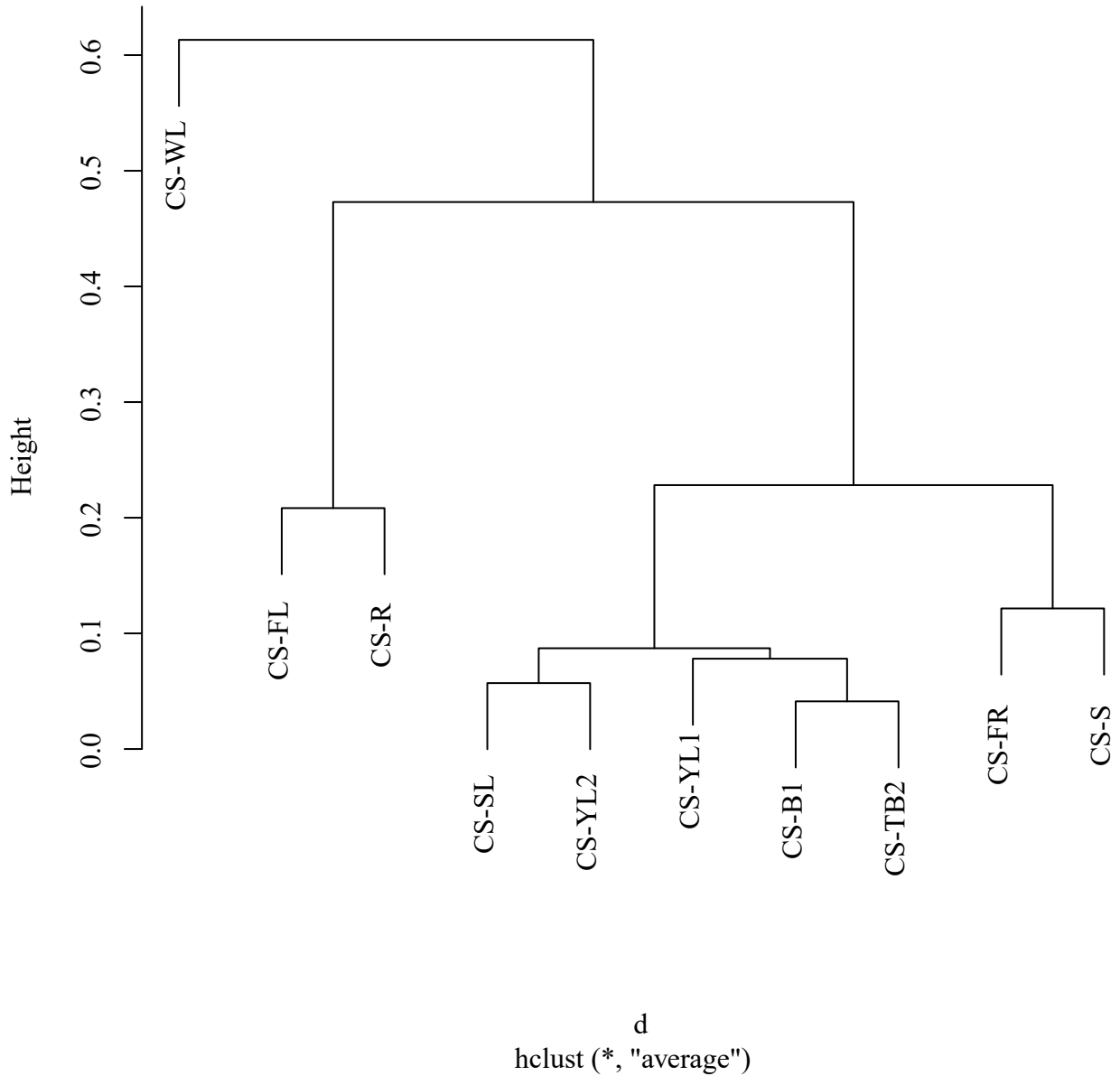

Supplement: Supplementary file 1 — Clustering dendrogram of samples based on gene expression. (PDF 99 kb) [file 12864_2018_4999_MOESM1_ESM.pdf]
